# Harnessing human and machine intelligence for planetary-level climate action

**DOI:** 10.1038/s44168-023-00056-3

**Published:** 2023-08-17

**Authors:** Ramit Debnath, Felix Creutzig, Benjamin K. Sovacool, Emily Shuckburgh

**Affiliations:** 1 Cambridge Zero and Department of Computer Science and Technology, University of Cambridge, Cambridge, CB3 0FD United Kingdom; 2 Division of Humanities and Social Science, California Institute of Technology, Pasadena, CA, 91125 USA; 3 Mercator Research Institute on Global Commons and Climate Change, Berlin, 10829 Germany; 4 Technical University Berlin, Berlin, 10827 Germany; 5 Center for Energy Technologies, Department of Business Development and Technology, Aarhus University, Aarhus, Denmark; 6 Science Policy Research Unit, University of Sussex Business School, Brighton, United Kingdom; 7 Institute for Global Sustainability, Department of Earth and Environment, Boston University, Boston, MA, USA

**Keywords:** Climate-change adaptation, Engineering

## Abstract

The ongoing global race for bigger and better artificial intelligence (AI) systems is expected to have a profound societal and environmental impact by altering job markets, disrupting business models, and enabling new governance and societal welfare structures that can affect global consensus for climate action pathways. However, the current AI systems are trained on biased datasets that could destabilize political agencies impacting climate change mitigation and adaptation decisions and compromise social stability, potentially leading to societal tipping events. Thus, the appropriate design of a less biased AI system that reflects both direct and indirect effects on societies and planetary challenges is a question of paramount importance. In this paper, we tackle the question of data-centric knowledge generation for climate action in ways that minimize biased AI. We argue for the need to co-align a less biased AI with an epistemic web on planetary health challenges for more trustworthy decision-making. A human-in-the-loop AI can be designed to align with three goals. First, it can contribute to a planetary epistemic web that supports climate action. Second, it can directly enable mitigation and adaptation interventions through knowledge of social tipping elements. Finally, it can reduce the data injustices associated with AI pretraining datasets.

## Introduction

The age of artificial intelligence (AI) has begun and is filled with opportunities and responsibilities. It is yet to be clearly understood how AI or machine intelligence can help address present global challenges, including climate change.

A global digital transformation would need an unprecedented level of machine intelligence. Making this machine intelligence sustainable and aligning it with planetary health challenges is a grand challenge on its own, starting with the rapid reduction of GHG emissions associated with the internet and currently carbon-intensive data centers^1,2^. The literature emphasizes several ways in which AI can play a crucial role in addressing climate change. It can provide innovative solutions to mitigate the negative impacts of greenhouse gas emissions, increase energy efficiency, and promote sustainable development^3^ (discussed later in detail).

Addressing climate change through AI is extremely challenging because of the enormous number of variables associated with this complex system. For instance, climate datasets are vast and take a significant amount of time to collect, analyze and use to make informed decisions that can translate into climate action. Using AI to account for the continually changing factors of climate change allows us to generate better-informed predictions about environmental changes, allowing us to deploy mitigation strategies earlier. This remains one of the most promising applications of AI in climate action planning. However, while explaining the potential of AI tools in physics-driven modeling of earth systems for predicting climate change, Irrgang et al.^4^ emphasize the need to rely on clear, physically meaningful research hypotheses, the geophysical determinism of process-based modeling and careful human evaluation against domain-specific knowledge to develop a meaningful AI that can address the challenges of climate science with classical earth system models.

Moreover, as the embodied impact of some of the current machine intelligence and AI systems associated with cryptocurrency mining, cloud computing, and large-scale machine learning models is just beginning to be understood, the accelerating impact of digitalization on consumption and resource extraction appears to be an increasingly troubling problem. As a result, our current trajectory of digitalization seems like a double-edged sword that may increase greenhouse gas emissions, worsening overall planetary health^2^.

Furthermore, digitalization’s influence on the natural environment and social systems is unknown and will require careful public policy design in many domains^5^. The desirable design of an accountable machine intelligence system, reflecting both direct and indirect effects on societies and planetary health, is a question of paramount importance that we expand on in this paper from a design thinking lens. We emphasize the need to co-align an epistemic web of planetary health challenges with the goals of a less-biased climate action AI, and debiasing large-scale pretraining datasets can pave the initial path. Key concepts and definitions used in this paper are illustrated in Box 1.

Box 1: Concepts and definitions **Machine intelligence** is the result of programming machines with some characteristics of human intelligence, such as learning, problem-solving, and prioritization, often enabled through machine learning (ML), which enables a machine to solve complex problems. ML is an application of AI that operates on deductive reasoning based on observed data, combining computation, models, and algorithms to make useful predictions or decisions.

True machine intelligence systems are envisaged to recognize when they have made mistakes, watch for similar data that could lead to a similar error in the future, and avoid repeating mistakes. Thus, it will have access to a variety of ML methods and automation techniques and will intelligently prioritize the achievement of specific objectives. In the present technological context, machine intelligence is defined as an advanced form of machine learning with the addition of prioritization and goals — a stepping stone on the road to true artificial general intelligence^74,75^.

We use the terms machine intelligence and AI interchangeably to represent our present digital age, which is expected to have profound societal and environmental impacts by altering the job market, business models, governance, and societal welfare structures^24^. However, a comprehensive understanding of desired and unwanted dynamic effects requires more attention, especially as bigger and better AI models are rapidly emerging.

**Digitalization** is the integration of digital technologies into societal structures and the economy. It is considered the backbone of the new machine intelligence—or AI—driven industrial age, called Industry 4.0 (and beyond). Not all forms of digitalization are positive, and evidence is emerging that digital devices such as smart meters or in-home displays and digitally connected or smart homes, could be more prone to increases in energy consumption and carbon emissions, vulnerability to hackers, invasions of privacy and surveillance capitalism, and even higher rates of domestic violence and abuse^76–79^.

**Planetary health challenges**^6^ include climate change, biodiversity loss, air pollution, water pollution, land use change, and food systems.

**The epistemic web** is envisaged by Jürgen Renn^8^, where the present internet (web) will become a universe of knowledge that parallels human knowledge. In the Epistemic Web, *browsing* will be replaced by the purposeful *federation* of documents. All data will be metadata, and all documents will be perspectives into the universe of knowledge. By allowing for greatly enriched links between documents (incoming as well as outbound links; multi-directional links; transitive and intransitive links; links with attached semantic labels; links with specified behaviors), the Epistemic Web will allow documents to describe one another. Thus, the digitization of current knowledge stores is essential; knowledge must be accessible, findable, and available for the recursive production of new knowledge.

**Human-in-the-loop AI** seeks to accomplish what neither humans nor machines can on their own. When a machine is incapable of solving a problem, humans must intervene. This procedure produces an ongoing feedback cycle. This AI design strategy is increasingly considered a way of creating a less biased AI.

**Social tipping points** (STPs) are instances in which a significant and rapid shift occurs in the attitudes, beliefs, behaviors, or values of an entire society or a substantial portion of it. These tipping points can be precipitated by a variety of factors, such as technological advancements, cultural movements, political events, economic shifts, or environmental crises^7,29,80–86^. These can lead to transformative changes in social norms, power and political institutions with long-lasting effects on individuals and societies as a whole. For instance, global climate strikes are viewed as social tipping events^81^.

These tipping points are shaped by social tipping elements (STEs) that include the energy production system, human settlements, the financial system, norms and values, the information system and the education system.

**Data justice** is defined as fairness in the way people are made visible, represented and treated as a result of their production of digital data, making it necessary to determine ethical paths through a datafying world^10^.

### An epistemic web of planetary health challenges for climate action

Climate action through machine intelligence must mean supporting climate mitigation and adaptation decisions at a global scale while avoiding excess emissions. However, the current generation of machine intelligence systems that drive digitalization has embedded biases and data justice issues, making them less trustworthy for transparent decision-making. Thus, for effective climate action, there is a need for a less-biased and collaborative AI that works not in competition with humans but with them to address such urgent planetary health challenges^6,7^ —emphasizing a human-centric/human-in-the-loop AI. Different people must bring their perspectives and knowledge to developing a less biased AI. Such a knowledge system could constitute what Jürgen Renn^8^ calls an ‘*epistemic web’*.

**Fig. 1:**
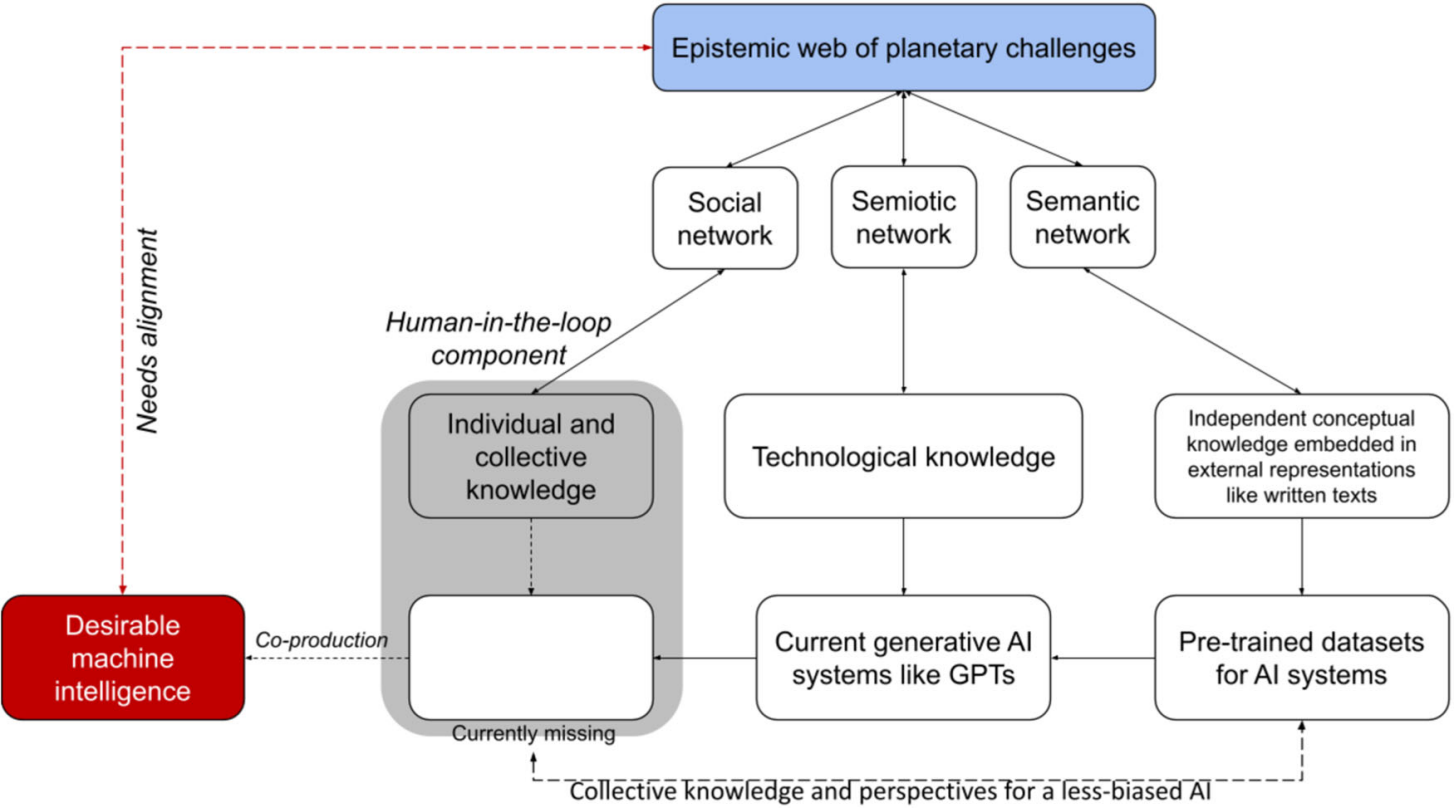
The need for co-aligning a less biased AI with a global epistemic web on planetary challenges. The epistemological basis is derived from ref. ^8^.

In this perspective, we investigate data-centric knowledge generation for climate action in the context of biased (or less biased) AI. For this, we envision the co-production of an epistemic web of planetary health challenges based on Renn’s epistemology^8^, relying on social, semiotic, and semantic networks for aligning desirable machine intelligence that captures the closely intertwined dimensions of the present human knowledge that informs current generation machine intelligence models (see Fig. 1). Individual or collective actors form the basis of a social network where these actors possess knowledge and are involved in producing, exchanging, disseminating, or appropriating knowledge. The process of social and communicative exchange manifests in the form of traditions, rules, conventions, and norms, as well as in terms of constraints and power structures that strengthen, weaken, facilitate, or impede ties within social networks^8^. These form the basis of the ‘contextualization’ of global challenges, where, for example, local knowledge and social norms can derive relevant climate mitigation and adaptation approaches^9^. However, existing data injustices represent a meaningful deterrent to realizing more inclusive knowledge and experience when it comes to climate action^1,10–12^.

The semiotic network, which communicates meaning, includes the entire material context of the action, including technological artifacts generated based on the technological knowledge of producers^8^. A recent example is OpenAI’s ChatGPT^13^, which uses billions of text parameters from Wikipedia and other internet sources as its pre-trained dataset. It produces ‘new knowledge’ in the form of a dialog format. According to Renn^8^, historically, semiotic networks are often the starting point for reconstructing other aspects of the epistemic web. This shapes the motivation of this article. As more significant and better AI models emerge, we can align accountable machine intelligence with an epistemic web of planetary health challenges.

Semantic networks must be reconstructed from external representations, such as written texts. It uses the fact that concepts have expressions in language. However, semantic networks have no one-to-one relation to either concepts or other cognitive building blocks; one and the same concept may correspond to dif­ferent terms in language, while the same term may represent dif­ferent concepts^8^. This has been the basis of many pre-trained large language models (LLMs) for AI systems, including foundational models like GPTs. Theoretically, Renn^8^ argues for deductively organized semantic networks to form highly organized knowledge systems. We expand Renn’s framework to create a way forward for co-producing a less biased machine intelligence with an AI-driven epistemic web of planetary challenges through digitalization actions.

**Fig. 2:**
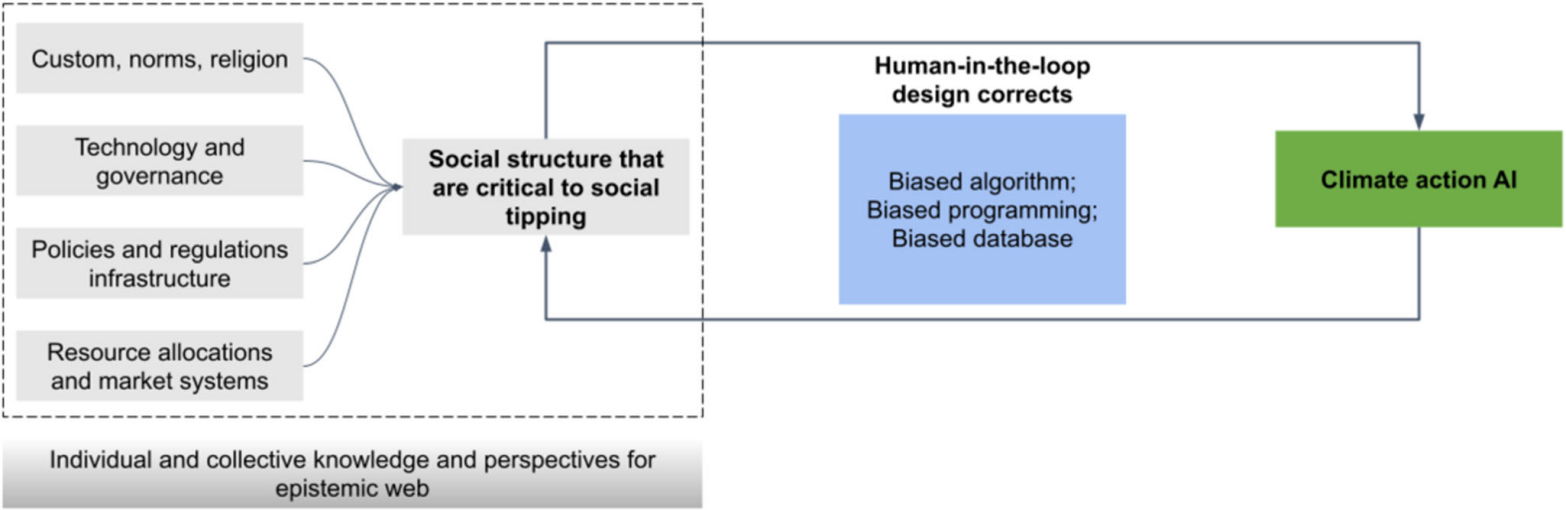
Envisioning a human-in-the-loop design of a climate action AI. Social structures and collective intelligence provide epistemic knowledge for climate action AI (Source: Authors).

**Fig. 3:**
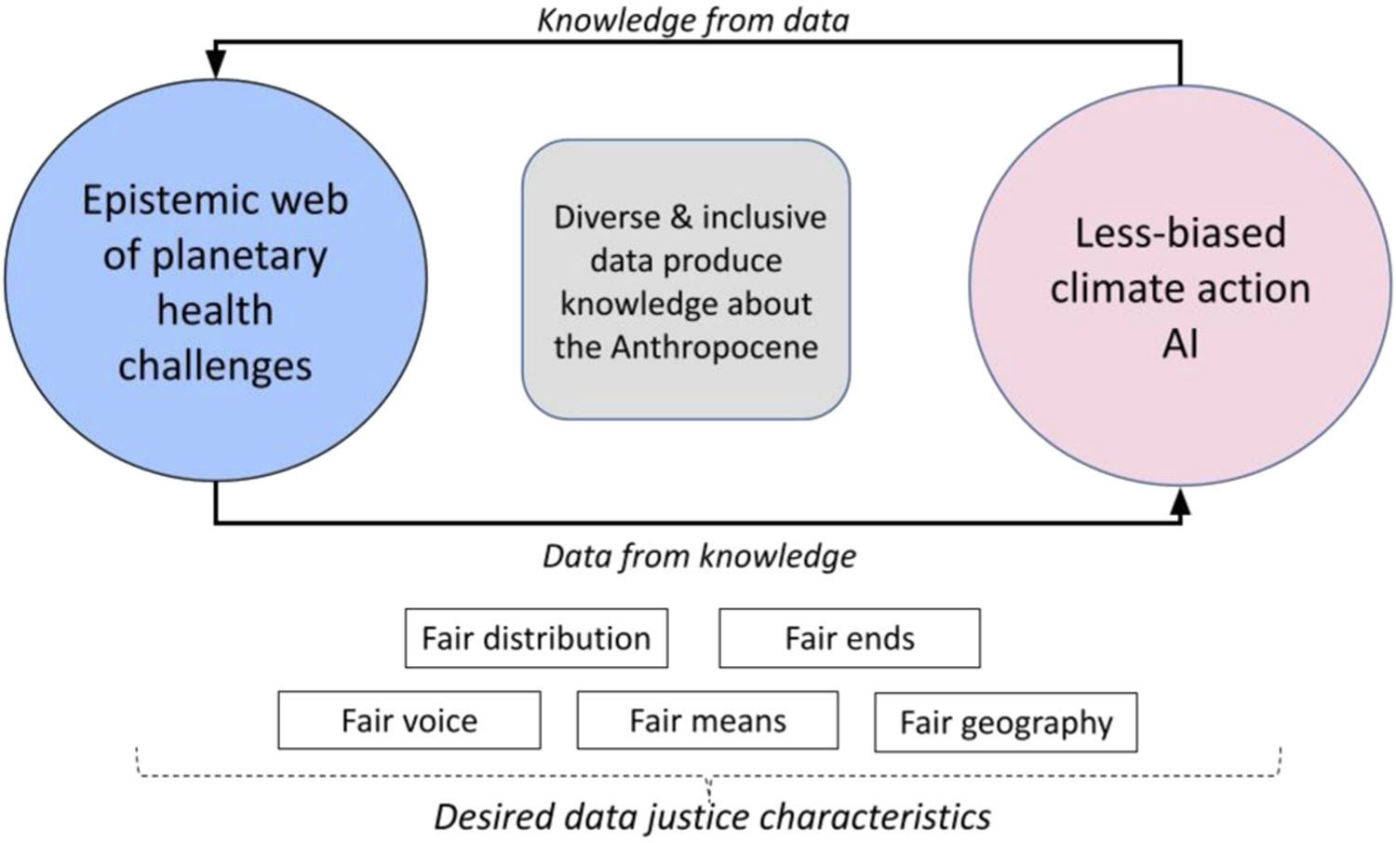
Data justice is essential for a climate action AI. Co-producing knowledge for an epistemic web of planetary challenges and less biased AI by leveraging desired data justice characteristics (Source: Authors).

Using the theoretical basis of the epistemic web of planetary health challenges, we take a deconstructivist approach to analyze how current pre-trained machine intelligence influences relationships among digitalization, equity, political agency, societal stability, and climate action. In doing so, we first define the scope of present machine intelligence systems in climate action, especially in relation to mitigation and adaptation. Next, we show that for an epistemic web of planetary challenges, there is a need to overcome accountability risks associated with biased AI systems for climate action (see Table 1). This is where the social network dimension of Renn’s epistemic web (see Fig. 1) becomes important as a foundation for collective consensus generation and societal stability. We emphasize that a human-in-the-loop AI design is critical to such an epistemic web that is free of biased datasets, biased programming, and biased algorithms (see Fig. 2). Finally, we emphasize removing the barriers to diversity and inclusion in the machine intelligence community to create grounded and reliable training datasets that can sustain an AI-driven epistemic loop of *‘data from knowledge’* and *‘knowledge from data’* (see Fig. 3).

**Table 1:** Fairness, Accountability and Transparency (FAccT) accountability risks with biased machine intelligence for climate action.

Sl no	FAccT accountability risks^17^	Implications for climate action AI design	Example scenario
1	Actor-related	Lack of contextualization of grounded reality associated with climate action (biased database). Therefore, the AI system cannot accurately and reliably predict the impacts of climate change and climate solutions in the near-term and long-term.	An AI model performs poor in terms of weather prediction due to lack of climate and earth system experts in the modeling team that code for physical mechanics. Similarly, an AI-driven climate mitigation model cannot represent social consequences of solutions due to misrepresentations of social scientists in the modeling team.
2	Forum-related	The machine intelligence model remains a black box system (a biased algorithm) mirroring hierarchical power relations and dependencies between different actors. This power structure impacts political accountability and decision-making.	Influential actor(s) obfuscate climate change information for political/business profitability gains through generation and amplification of misinformation, leading to polarization and political manipulations. This can potentially destabilize democracy through political instability.
3	Relationship-related	Biased databases cause a lack of diverse knowledge and perspectives in climate AI models, making them skewed towards specific outcomes/groups.	Policymakers rely on an AI system to design emission reduction scenarios in a Global South city without consideration of climate change-induced migration and urban sprawl. Similarly, a lack of indigenous knowledge in the AI system systematically excludes local communities from biodiversity conservation decision-making. Inaccurate climate modeling and forecasting due to data biases.
4	Account-related	Biased programming makes it harder to differentiate which actor should account for what, leading to incompleteness in decision-making.	The climate AI system predicts extreme weather impacts for Global North countries with 98% accuracy, while for Global South countries its accuracy is set at 60%.
5	Consequence-related	Lack of regulation and procedural measures allows the climate AI industry to build megasystems for specific groups that imbalance political accountability and power structures.	Data privacy is breached using an AI system for millions of people to track their sustainability behavior and enforce climate inaction penalties.

### Machine intelligence accountability risks for climate action

At present, machine intelligence systems for climate action are at an early stage of development, and their impact is just beginning to be understood, which embeds biases in their entire value chain, making these AI systems less trustworthy for climate action decision-making^14^. Drawing inference from the application of AI in climate modeling (as discussed above), biases can influence prediction accuracy, reliability, and interpretability, which can seriously affect decisions for climate mitigation and adaptation actions. For example, if a biased climate model is trained on data that excludes certain regions or time periods, the predictions may not accurately reflect the complete scope of climate change. Similarly, such models may underrepresent certain variables or factors and provide inaccurate estimations of carbon emissions from particular industries, leading to an underestimation of the actual impact of those variables on the climate. Furthermore, biased climate models can worsen climate impact and response inequities. If AI models only consider how climate change will affect a small number of regions or populations, those regions and people may experience disproportionately negative effects.

Biases can arise from overlapping classes like biased databases, biased algorithms, and biased programming. For instance, Rich and Gureckis^15^ point out three causes of bias in present machine intelligence systems: small and incomplete datasets, learning from the results of our decisions, and biased inference and evaluation processes. These biases reduce the accuracy and reliability of many present-generation machine intelligence systems for climate action, making them less accountable, interpretable, and explainable (see a comprehensive survey of black box models here^16^). Fairness, Accountability, and Transparency (FAccT) researchers present five algorithmic accountability risks^17^ that can emerge from such biases. We synthesize these risks with respect to the design of climate action AI (see Table 1).

While FAccT and AI ethics researchers are beginning to discuss the potential role of AI in mitigating the aforementioned accountability issues in climate action^3,14,18^, the paths to developing a less biased AI for climate assessment remain uncertain. From this paper’s scoping of the epistemic web in Fig. 1, we focus on the need for quality training datasets that represent the diverse grounded reality of human perspective and experiences (epistemic knowledge) with as little bias as possible, which becomes highly critical in making AI less biased^19,20^. Also, this feature of representing the different kinds of human knowledge in the machine intelligence system is needed when these models will be relied upon to make decisions about how to deal with climate change as a planetary health challenge. For example, Gupta et al.^21^ define and implement Earth system justice to link the physical and social aspects of climate change and make sure that planetary health actions reduce harm, improve well-being, and reflect both substantive and procedural justice.

Thus, a desirable feature of machine intelligence and AI systems for climate action is the embedding of epistemic knowledge, which can be achieved through diverse and representative pre-training datasets. However, literature shows embedding epistemic knowledge is not simple, as even the most advanced present generation AI systems must be made more transparent, explainable, and interpretable^22^. Interpretability means that cause and effect can be determined in machine learning (ML) models. Explainability is crucial for piercing the black box of ML models and understanding how the model works^16,23^. These two characteristics are critical for reducing algorithmic accountability risks (see Table 1) and making AI safer for high-stakes climate action decision-making^23^.

Another significant impediment is the need for more precise uncertainty quantification in existing AI systems^24^. It makes many current-generation machine intelligence systems overconfident, and they make mistakes^25^. Therefore, the current epistemic base (like the world wide web) for machine intelligence embeds these limitations and biases that make it less practical for individual and collective decision-making for climate action. As a result, current AI systems are less useful for direct applications in climate mitigation and adaptation.

### Aligning human-in-the-loop AI design with climate action

Recent advances in human-in-the-loop machine learning approaches using large-language models (LLMs) show a way forward to integrate epistemic feedback loops into black box models. Human-in-the-loop models refer to machine learning systems or algorithms that involve human interaction and input to improve their accuracy. A recent example is Open AI’s ChatGPT^13^ which uses a human-in-the-loop^26^ system. In ChatGPT, the AI system interacts conversationally with the human user. This dialog format makes it possible to answer follow-up questions, admit mistakes, challenge incorrect premises, and reject inappropriate requests^13^. The machine intelligence element of ChatGPT is in its model training using reinforcement learning from human feedback (RLHF)^27^ driven by the proximal policy optimization (PPO) algorithm.

As a starting point for conceptualizing an epistemic web, Creutzig et al.^2^ demonstrate a relationship among digitalization, equity, political agency, climate change and planetary stability. It emphasizes AI’s direct and indirect impact on climate action. For example, a direct impact like energy demand for training large machine learning models in data centers. Indirect impacts like machine intelligence applications that reduce greenhouse gas emissions and environmental impact. The digitalization of social networks via algorithms (i.e., social media platforms) is instrumental in creating polarization (through misinformation and disinformation)^28^ and shaping political opinion that affects social equity within and between countries. High levels of inequity and polarization reduce the feasibility of consensual climate actions, leading to irreversible social tipping scenarios. They are thus indirectly relevant for machine intelligence design and its reward models.

We connect the epistemic interdependencies of machine intelligence with political agency and democracy, equity, and social tipping elements (discussed in the next section). We illustrate an epistemic web basis to define desirable machine intelligence for digitalization that balances social equity, stabilizes political agency (and therefore democracy), and ensures climate mitigation and adaptation goals are met through sustained climate action, thus, potentially preventing irreversible social tipping^3,29,30^. Digitalization should enable collective action (as data for knowledge) to be transferred from the epistemic web of planetary challenges for training the AI systems. Thus, enabling knowledge generation from the data. This will define the true scale of a human-in-the-loop system for planetary-level digitalization.

Increasingly, the context of human-in-the-loop AI is gaining critical importance, as it is beneficial to reduce biases when a diverse group of humans with different identities, backgrounds, and viewpoints, using collective intelligence^31^, participate in machine intelligence system design^26^. Under the best circumstances, utilizing such collective intelligence for human-machine collaboration and co-creation results in knowledge generation as an epistemic web.

We present this design framework in Fig. 2, which emphasizes that the epistemic web contains grounded and diverse knowledge of social structures that are critical social tipping elements^29^. In the social epistemic network (see Fig. 1), AI systems trained through such an epistemic web can help reduce misinformation, remove skepticism, and restore trust^32^. Thereby ensuring the stability of socio-political institutions that are critical for determining consensus for climate action.

When envisioning a climate action AI system, we establish that such systems are driven by the need for accountability risk reduction (see Table 2) that delivers a less biased AI, coupled with the drive for planetary-scale digitalization that enables collective climate action, which by itself is influenced by the epistemic web. This epistemic web creates a more robust foundation for collective decision-making and individual action, efforts that could come to play a more important role in accelerating climate policies for mitigation and adaptation that could contribute towards minimizing the risks of irreversible social collapse.

**Table 2:** Human-in-the-loop design for improving the scope of climate action AI.

Scope	Potential benefit from AI	State-of-the-art machine intelligence approaches	Examples from industry	Scope for human-in-the-loop climate action AI design
Climate modeling	More accurate climate models can be created by analyzing data from a variety of sources, such as satellite imagery and weather stations, improving our understanding of the Earth’s climate and predicting future changes.	Deep learning-driven Generative Adversarial Networks (GANs); Reinforcement learning; Gaussian process regression; Bayesian inference; Convolutional Neural Networks (CNNs); Ensemble learning; Transformer-based model (also called ‘Foundation models’)	Deepmind is using GANs for improving nowcasting of rain in the UK^54^. IBM Global High-Resolution Atmospheric Forecasting System (IBM GRAF) uses ensemble learning approaches for weather and solar forecasting^55^. Google uses a 2-D and 3-D CNN called U-Net for precipitation nowcasting using radar images^56^. Microsoft is building a planetary computer.	Intelligent Integrated Assessment Models that capture the grounded realities of climate vulnerabilities and impacts.
Energy management	Optimize energy management systems in buildings, factories, transportation systems and power grid. AI can aid in improving safety, energy efficiency, reducing energy waste, and help save costs by analyzing multimodal data on energy demand, weather patterns, and user behavior.	Convolutional Neural Networks (CNNs); Deep Neural Network; Deep Reinforcement Learning; Explainable AI (XAI) techniques like General Additive Models (GAM); Local Interpretable Model-agnostic Explanations (LIME); Shapley Additive Explanations (SHAP); Transformer-based Model (also called ‘Foundation models’); Explainable AI (XAI) techniques	ABB Ltd. (Switzerland) using computer vision and CNN for faults in energy pipelines.	Better energy demand forecasting, energy poverty and vulnerability identification, and robust distributive and procedural energy resource optimization lead to improved emission reduction strategies and enactments.
Renewable Energy	Accelerate integration of renewable energy sources like solar and wind power into the electric grid. It can optimize power generation, storage, and distribution at-scale by forecasting energy demand and adapting energy supply to meet it in real-time.	Convolutional Neural Networks (CNNs); Deep Neural Network; Generative Adversarial Networks (GANs); Meta-heuristic algorithm and random forests; Reinforcement Learning	Open Climate Fix (UK) is using CNN for near-term forecasts of Solar Energy for UK Grid. GE Renewable Energy (USA) is using various AI techniques for health monitoring and asset management of its wind farms.	Improved grid flexibility, market resilience, and cost effectiveness lead to greater uptake of renewables at a national, regional, and local scale, fulfilling emissions reduction targets. Human-in-the-loop technology enables better decision-making for targeted applications.
Climate adaptation	Support climate adaptation efforts by predicting the impacts of climate change on different ecosystems and populations, enabling policymakers and planners to develop more effective strategies to protect vulnerable communities and infrastructure.	Convolutional Neural Networks (CNNs); Deep Neural Network; Explainable AI (XAI) techniques like Layer-wise Relevance Propagation (LRP), General Additive Models (GAM), Local Interpretable Model-agnostic Explanations (LIME); Counterfactuals and Rationalization.	ClimateAi (USA) provides long-term and short-term climate risk assessment. One Concerns (USA) uses AI to estimate damage from natural phenomena. Prospera (Tel Aviv) uses computer vision to monitor and analyze plant health and stress using multi-layer crop field and climatic data. EXCI (Australia) uses data fusion techniques from satellites and ground-based sensors for bushfire detection.	Local knowledge and traditional climate resilience building techniques help AI develop robust adaptation techniques. Also inherently debaises the AI with representative datasets.
Carbon Capture	Help improve the efficiency of carbon capture and storage technologies by analyzing data on carbon dioxide emissions and identifying the most optimized ways to capture and store it.	Convolutional Neural Networks (CNNs); Deep Neural Network; Fourier Neural Operators (FNOs); Explainable AI (XAI) techniques like: explainable neural network (XNN), local interpretable model agnostic explanation (LIME), shapley additive exPlanations (SHAP), Deep Learning Important FeaTures (DeepLIFT)	NVIDIA (USA) and Northern Light Initiative (Norway) created U-FNOs for simulating pressure levels as multiphase flows during carbon storage.	Better understanding of the unintended socio-political and economic consequences of carbon capture and climate engineering applications. An AI trained with public perceptions of such technologies can iterate hundreds of contextualized emission reduction scenarios that can help deliver consensus for technology-driven climate action.
Cities	Help monitor emissions and support optimal urban planning and infrastructure provision in anticipation of induced energy use and GHG emissions.	Explainable AI approaches to explain how urban form features induce transport GHG emissions, XGBoost for estimating building properties	Various attempts to create digital twins of cities.	Co-creation of urban design interventions using people’s lived experiences of cities leads to hyperlocal emission reduction and adaptation solutions. Such solutions can have a long-lasting impact on city-level climate action.
Collective intelligence	Help aggregate collective and individual voices for understanding social agencies, norms and customs that shape socio-political institutions responsible for climate action consensus.	Foundational models like BERT, GPTs.; Random forests; XGboost; Computational Social Science approaches	Early stages, mostly academic research; Climate Assembly UK; Environmental Justice Index by Center for Disease Control, USA. ClimateBERT uses fine-tuned large language model for making climate Risk understandable and climate information more accessible to the broader communities.	Help counter misinformation, polarization, and eventually political instability that could affect consensus for climate mitigation and adaptation. Aggregating large-scale public voices on various climate change impact issues can also create an inclusive and diverse pre-training data infrastructure for generative AI systems.

Source: Authors, compiled from refs. ^3,14,18,32,39,57–62^.

### Human-in-the-loop AI designed on social tipping points

The most pressing challenge in this context is the need for more diverse and reliable datasets to build different and reliable algorithms that represent grounded reality, as well as deliberate decision-making on these algorithms, which is shaping current debates on the urgent need for data justice and its agencies^10^.

For example, Schramowski et al.^33^ have shown that large language models (LLMs) such as BERT, GPT-2, and GPT-3 trained on unfiltered text corpora suffer from degenerated and biased behavior. Nonetheless, the authors successfully demonstrated that the human-corrected, pre-trained LLM could mitigate the associated risks of toxic degeneration. They used a questionnaire survey of 117 questions to create a human-in-the-loop design to give the AI system a moral direction of what is right and wrong to do. This characteristic of climate action AI systems is critical to shaping the epistemic web that embeds knowledge layers of social structures critical to social tipping points.

Such interactive learning of machine intelligence with human intelligence is desirable to foster AI-driven climate action. This human-machine interactivity is at the core of accountable AI systems^34^ that can reason about social, cultural and moral norms as critical social structure datasets for enabling climate mitigation and adaptation consensus which do not exist currently^35^. An attempt was made by Forbes et al.^36^ through the creation of a large-scale social corpora called Social-Chem-101. Similarly, Colas et al.^37^ conceptualized the immersion of autotelic agents into rich sociocultural worlds for making AI systems more trustworthy (Autotelic agents are intrinsically motivated learning agents that can learn to represent, generate, select and solve their own problems).

As a timely case study for generative AI, reinforcement learning through human feedback (RLHF) in ChatGPT shows that human intelligence can be integrated with machine intelligence to design specific reward models for the AI system. This can be leveraged to improve the trustworthiness of machine intelligence systems through a human-centered design approach^38^ for fine-tuning RLHF that asks what is desirable in the current megatrend of digital transformation of economies and societies. Such applications of human-in-the-loop design show opportunities for contextualizing machine intelligence for system-scale behavioral climate action that prevents social tipping points (STPs, see Box 1)^32^. In Box 2, we present a theoretical scoping of data representing social structural layers of social tipping elements (STEs).

It is not yet known how LLM-based human-in-the-loop AI systems like ChatGPT can support climate mitigation and adaptation, especially across the STEs. One recent example is the creation of ClimateBERT, where researchers fine-tuned a LLM using the IPCC reports for improving public understanding of climate risks and making climate information more accessible to the wider community^39^. However, caution should be taken to remove existing biases and uncertainties in machine intelligence design and operations. For instance, researchers at DeepMind recently unveiled a taxonomy of risks posed by LLMs, which are used to train generative AI^40^, including: i) discrimination, hate speech, and exclusion; ii) information hazards; iii) misinformation harms; iv) malicious uses; v) human-computer interaction harms; and vi) environmental and socio-economic harms. A part of this problem is that such LLM-based AI systems are far from trustworthy AI systems, as their interpretability and explainability are still exclusively dependent on their pre-training datasets from internet sources like Wikipedia, News, and BookCorpus databases. This triangulates our focus on the need to align with an epistemic web that represents reliable and diverse human knowledge and perspectives.

Box 2: Societal structure data layers in social tipping elements for climate action AI applications

**Table:** 

**Application areas**	
Climate Modeling	Knowledge of custom, norms, religion, political institutions, individual and group identity shapes consensus for mitigation and adaptation actions using AI.
Energy Management	Reflect human behavioral attributes of energy consumption at scale and its contextualization with energy justice dimensions.
Renewable Energy	Data concerning production and demand forecasting, grid stability and market allocations at a granular and socio-economic interactive scale.
Climate adaptation	Epistemic knowledge representation from vulnerable and marginal communities improves the diversity and inclusivity of climate impact datasets.
Carbon capture	Socio-economic data on carbon offset impacts across local, national and regional scales.
Cities	Large-scale behavioral datasets about urban adaptation and resilience strategies grounded in localized knowledge of customs, culture and norms.
Collective intelligence	Information about the socio-political consensus drivers for climate action, including countering mechanisms for dis- and misinformation, polarization across cultures and norms.

### Co-producing knowledge for climate action and a less biased AI

Present-day AI is less trustworthy for decision-making, especially when it relates to climate mitigation and adaptation efforts. We synthesized the accountability risks associated with such systems in Table 2, as well as the need to act on biased programming, biased algorithms, and biased datasets for a more trustworthy climate action AI. For example, in the previous section, we emphasized that biased AI-led climate modeling can lead to inaccurate forecasting and impact assessment, which will affect decision-making. To correct such biases, the AI systems require humans-in-the-loop, especially to produce and feed the training data into the algorithms at the initial stage of model development^26^. This calls for sincere efforts towards embedding *data justice* in the pretraining datasets.

In this purview, we argue that a human-in-the-loop design of the climate action AI is critical that embraces diversity in perspectives and knowledge from engineers, social scientists, philosophers, industry practitioners, policymakers and the public^26^. For example, the concept of trustworthy AI that is humanistic, just, and ethical is at the core of a desirable machine intelligence system’s design^19^. We expand this argument for a human-in-the-loop climate action AI.

However, the notion of algorithmic trust is subjective to the context (as illustrated in Table 2) and emphasizes the need for the ML/AI experts to relate how their metrics of trust impact trust by individuals and the public^41,42^. This makes fairness-aware machine learning (ML) a difficult challenge as the measurement parameters of fairness in terms of bias and related notions are still not clearly understood. For instance, Narayanan^43^ has identified 21 definitions of fairness in the literature, which cannot necessarily all be obtained at the same time.

Two widely used definitions that have been widely incorporated into ML pipelines are those of *individual fairness*, which states that individuals who are similar should be treated similarly, and, *group fairness*, which states that demographic groups should, on the whole, receive similar decisions^44,45^. It is important to understand what assumptions are reasonable in a given context before developing and deploying fair mechanisms (i.e., contextualization); without this work, incorrect assumptions could lead to unfair mechanisms^44,46^.

Bridging such an epistemic gap associated with the meaning of fairness is critical if climate action AI systems are to design and implement climate mitigation and adaptation strategies. FAccT scholars have proposed various solutions to establish a subset of fairness notions^41,42,47^. One approach that is most relevant to our paper’s scoping is to reduce the bias in the pre-training dataset, known as *debiasing data*, which can fulfill the objectives of data justice^10^.

A critical step towards debiasing pretraining datasets is creating data-centric AI that emphasizes more accountable and context-specific datasets related to climate action. FAccT literature stresses the need for ‘social transparency’ in making AI systems trustworthy by aligning efforts needed to establish organizational and regulatory ecosystems for the assurance of trustworthy AI in the public domain (e.g., the MATCH model)^48–50^. Moreover, literature at the intersection of social science and data science shows that a data justice lens is instrumental in bringing social transparency (which improves trustworthiness)^10–12^.

In Fig. 3, we highlight that for debiasing the pretraining datasets of a climate action AI, we must create a self-sustaining and interactive mechanism of *‘data from knowledge*’ and ‘*knowledge from data*’. The ‘data’ in this case must contain multi-layered information on climate change impacts, mitigation, and adaptation strategies at an anthropocene scale, which can then generate the needed ‘knowledge’ base for appropriate and contextualized climate action for the avoidance of irreversible social tipping, as discussed earlier.

Here, we synthesize the desired data justice characteristics of a less biased climate action AI that embeds the knowledge of societal structural layers (see Fig. 2) by leveraging existing social justice dimensions of instrumentality (fair ends), distribution (fair allocation), and procedure (fair means) that correspond to the data diversity and inclusion needs through recognition, ownership, structure and space, as illustrated in Table 3. For example, a lack of data justice and its contextualization in most climate-vulnerable regions of the world pose a significant risk of training a biased AI that can virtually hallucinate during decision-making applications. We are already experiencing hallucinatory results with ChatGPT.

**Table 3:** Data justice characteristics for more equitable climate action AI.

Social justice characteristic	Application to data justice for a less biased human-in-the-loop AI		Implications for a climate action AI design
	Injustice issues	Justice measures	
Instrumental (fair ends)	Inequitable distribution of the benefits or risks of data sharing or AI programming.	Guaranteeing the rewards of AI reduces lessen rather than worsen economic and social vulnerability.	Inclusive and diverse pre-training datasets create more accurate and efficient climate models that can help in better vulnerability estimation, leading to improved climate mitigation and adaptation planning at urban, regional and national scales.
Procedural (fair means)	Exclusion of key stakeholders from data discussions and obfuscation of biases within AI programming.	Ensuring that individuals and groups have a meaningful voice in the design and operation of AI systems, including the right to consent (or withdraw consent) for the use of their personal data.	Effective consensus generation for climate action by leveraging diverse and context-specific collective intelligence. Improved renewable energy forecasting and energy management in a changing weather context that promotes energy justice in vulnerable areas.
Distributive (fair distribution)	Consolidation of intellectual property and big data among corporate or technology elites	Maintaining open and accessible data, and community based ownership platforms (e.g., cooperatives, community interest companies) and prioritizing minority or vulnerable groups in digitalization processes and outcomes.	Better identification of climate vulnerability groups and their social structures, resulting in better mitigation and adaptation measures across diverse population groups. Enables robust decision-making and accurate estimation of the distributive impact of disruptive climate technologies associated with carbon capture and storage.
Recognition (fair voice)	Discrimination against particular demographic or indigenous groups and failure to respect privacy	Including groups in design and governance of data systems. Providing stronger accountability measures.	Enables collective intelligence at the pre-training stage, which can embed valuable grounded knowledge and perceptions of climate change impacts. It further strengthens an epistemic web of planetary health challenges. Climate models become more grounded and context-driven, leading to better forecasting and disaster preparedness.
Spatial justice (fair geography)	Uneven siting and location of digital infrastructure, as well as uneven access to data.	Providing access to digital infrastructure and resources as a common pool and open resource rather than a restricted product, improving accessibility for all stakeholders.	Climate models become spatially effective across varying granularities. It enables better mitigation and adaptation planning in vulnerable areas, as well as improved disaster resilience. Collective intelligence gets strengthened and streamlined due to the shrinkage of the global digital divide. Cities are planned with greater emission reduction potential. Social structures are made more resilient through diverse and inclusive people-centric datasets, leading to the efficient use of collective intelligence. Better energy management and renewable energy forecasting across geographical scales will lead to improved accountability for emission reduction actions.

Source: Authors’ synthesis from refs. ^10–12,63–73^.

The social justice characteristics and references in this table is based in work comparing data justice with energy justice done originally by Max Lacey-Barnacle and Adrian Smith as part of the Responsive Organizing for Low Emission Societies (Economic and Social Research Council Grant Agreement No. ES/V01403X/1), and which we have adapted and modified for this paper.

In Table 3, we connect these characteristics to the specific requirements of a less biased climate action AI that have applications in climate modeling, energy management, city planning, carbon capture and storage, and collective intelligence (as illustrated in Box 2).

Researchers are finding innovative ways to produce data-centric infrastructure to support this goal. For example, African AI researchers have established a common AI dataset repository for their local context, COCO-Africa^51^. MasakhaNER provides a large curated dataset of ten African languages with named-entity annotations^52^. Such initiatives are still very early, and more effort is needed to mainstream them, especially along the five data justice characteristics levers.

Without diversity and the inclusion of a full range of populations, we risk the development of biased algorithms^20^ and, subsequently, a biased epistemic web. Moreover, there is an added risk of failing to fulfill the AI talent pool and missing its broader societal benefit towards solving planetary challenges like climate change, as discussed above. Using AI for climate action (mitigation and adaptation) is especially challenging as it can be a double-edged sword that may increase greenhouse gas emissions and worsen overall planetary health, which we discuss from a social tipping point lens. This effect is due to embedded biases and injustices in the training datasets used in the design of present-day generative AI systems.

## Conclusion

We conceptualized the co-production of an epistemic web of planetary challenges based on Renn’s epistemology^8^ for aligning desirable machine intelligence that captures the closely intertwined social, semiotic, and semantic network dimensions of present human knowledge that inform current generation pre-trained AI models. This epistemic web can help reduce accountability risks^17^ associated with machine learning and AI models while correcting algorithm-driven polarization for climate action^32^, leading to collective consensus for climate adaptation and mitigation policies. We envisaged leveraging the recent advances in human-in-the-loop AI through political agencies and democratic decision-making^2^. However, existing embedded inequalities associated with AI system fairness, ethics, and biases must be addressed.

The need of the hour is to be sensitive to digital inequalities and injustices within the machine intelligence community, especially when AI is used as an instrument for addressing planetary health challenges like climate change. That is where the role of social science, philosophy and the humanities becomes even more critical. A recent AI community review^53^ touched on this theme, specifically focusing on academic data science researchers. Bridging such a divide and debiasing datasets should be a core component of a desirable machine intelligence-driven digitalization system. Otherwise, the pre-trained datasets will remain biased and overrepresent certain groups. Thus, leading to a biased climate action AI.

Similarly, there is strong evidence of structural inequalities in climate action (mitigation and adaptation) between and within countries. It is even more prominent in vulnerable and resource-constrained communities in the Global South. Such inequalities, if sustained, are estimated to have catastrophic outcomes impacting societal collapse and planetary stability, including not fulfilling any climate mitigation pathways^29^.

Better aligning less-biased AI with climate protection and respecting planetary boundaries also creates an unprecedented opportunity to act on global injustices and embed positive data justice thinking in the current wave of digitalization. This encompasses ensuring that the benefits of digitalization redress existing injustices, that vulnerable groups are more involved in standard-setting and policymaking and that disadvantaged groups, in particular, have access to open data. It also suggests that ownership and exploitation of data expand to include civil society and communities themselves (e.g., via cooperatives and trust arrangements), the active participation of users, and the promotion of broadband internet access as a public good rather than a private commodity.

As machine intelligence increasingly impacts society, diversity and inclusion are growing concerns. Therefore, the co-creation and co-production of relevant knowledge, infrastructure, and human resources must be a desirable machine intelligence design priority that defines an epistemic web of collective action for addressing planetary challenges.

### Reporting summary

Further information on research design is available in the Nature Research Reporting Summary linked to this article.

### Supplementary information

Reporting Summary
